# A Tailored Nurse Coaching Intervention for Oral Chemotherapy Adherence

**Published:** 2014-05-01

**Authors:** Susan M. Schneider, Donna B. Adams, Tracy Gosselin

**Affiliations:** Duke University School of Nursing and Duke University Health System, Durham, North Carolina

## Abstract

Although patients frequently express a preference for oral medications, compliance to these medications varies. Patients often have difficulty adhering to their medication schedules due to lack of understanding, inadequate support, or unwelcome side effects. Fostering adherence to oral chemotherapy regimens improves patients’ chance of survival and long-term quality of life. This randomized trial tested the effectiveness of a tailored intervention to promote adherence to oral chemotherapeutic agents in 45 adult patients with cancer. The control group received the standard chemotherapy education provided at the cancer center. The intervention group received standard education and a tailored adherence plan developed by an advanced practice nurse. The nurse coaching intervention was administered via telephone. Adherence was measured using self-report and pharmacy refill rates. For adherence measures at both 2 and 4 months, the intervention group adherence rates were superior to the control group rates. Pharmacy refill rates of adherence were lower than self-reports. Results suggest that for some participants, the tailored coaching intervention was bene-ficial. Barriers to and facilitators of better adherence are discussed.

Oral chemotherapy is prescribed to diminish tumor mass, eradicate micrometastatic disease, and increase disease-free survival in cancer patients. Although cancer patients frequently express a preference for oral medications, compliance to these medications varies. Patients often have difficulty adhering to the prescribed schedule because of a lack of understanding, inadequate social support, or treatment-related side effects, among other factors. Patients who successfully adhere to chemotherapy regimens have a greater chance of nonrecurrence and long-term quality of life. Thus, helping patients tolerate oral chemotherapy regimens is critical to their survival.

The purposes of this study were (1) to test the effectiveness of a tailored protocol to promote adherence to oral chemotherapeutic agents in adults receiving treatment for cancer, and (2) to explore whether age, gender, and depression affect adherence to oral chemotherapeutic agents. Nursing interventions designed to promote adherence can help to enhance chances for cure and improve patient quality of life. Using advanced practice nurses (APNs) to test the effectiveness of a theoretically based intervention can lead to an evidence-based strategy that can be routinely implemented by clinical nurses.

## Background

Several Institute of Medicine (IOM) reports have focused on patient-centered care. The report entitled *Crossing the Quality Chasm* (IOM, 2001) recommended a change from provider locus of control to patient locus of control (Berwick, 2009). The IOM report described the concept of patient-centeredness as "care that is respectful of and responsive to individual patient preferences, needs and values, and ensuring that the patient values guide all clinical decisions" (IOM, 2001, p. 6). This report suggested that the relationship between the provider and the patient is the foundation to promoting individual participation regarding treatment decisions. Recognizing that the illness experience varies among individuals and changes throughout the course of treatment, providers must be flexible.

Chemotherapy has traditionally been administered intravenously in the hospital or outpatient infusion center. These settings allow for strict control of dosage and assure that patients receive their prescribed treatments. During infusions, patients are closely monitored for side effects, education is provided, and nurses are available to answer questions and provide support (Moore, 2007). However, even in these controlled settings, patients can have difficulty adhering to their regimens because of a lack of understanding, inadequate support, and unwelcome related side effects (Dodd, Miaskowski, & Paul, 2001; Schneider, Hess, & Gosselin, 2011).

Oral cancer therapies have several advantages, including greater flexibility and convenience for the patient and minimal disruption of the activities of daily living. Patients report a preference for oral chemotherapy over infusional therapy (Liu, Franssen, Fitch, & Warner, 1997; Twelves, Gollins, Grieve, & Samuel, 2006), most often citing the fact that oral chemotherapy does not require IV access or additional visits to the clinic/hospital for treatment as the reason for this preference.

Yet oral therapies do have disadvantages. The first is cost: Patients may have high copayments depending on their health insurance plans; patients on fixed incomes may not be able to afford the additional expense. In addition, the side effects of these agents are similar to those of IV agents, and new side effects such as skin rashes may also occur (Given, Spoelstra, & Grant, 2011). Patients may decrease or skip doses if the side effects are too troubling, and as a result, they may not receive the recommended dose.

Systematic reviews focusing on adherence to antineoplastic agents report rates ranging from 16% to 100% (Escalada & Griffiths, 2006; Ruddy, Mayer, & Partridge, 2009). Adherence rates can vary depending on the complexity of the medication regimen (Given et al., 2011). Inadequate adherence can result in a disruption of the therapeutic index needed to eradicate the cancer. Hence, it is critical to develop individualized interventions that encourage patients to adhere to oral cancer chemotherapy and thus increase their chances of survival.

As patients become better consumers and advocates for their own care, health-care providers need to be aware of how their behaviors and communication patterns affect adherence. Provision of information is the cornerstone of patient empowerment. Thus, educational as well as behavioral strategies need to be incorporated into the treatment plan (Balkrishnan, 2005). Nursing interventions have been shown to have positive effects on adherence as well as symptom management (Foley, D’Amico, & Merenstein, 1995; McCauley, Bixby, & Naylor, 2006; Vied, Caron, Rosenthal, & Weismantel, 2004). However, because of lifestyle variations, not all interventions work with all patients. Interventions need to be tailored to individual needs (Schneider et al., 2011).

The tailored coaching intervention used in this study identified individual barriers to and facilitators of taking oral chemotherapy and worked with patients to identify strategies that helped individuals take their medications as prescribed. The initial phase of the intervention protocol involved the assessment of barriers to and facilitators of adherence. An APN used instruments and interview questions to identify factors such as gender, cognitive function, presence of a caregiver, and complexity of the medication regimen, all of which can influence adherence. Then, based on the results of this assessment, strategies to improve patient knowledge, enhance behavioral skills, and strengthen affective support were implemented. The intervention was based on the Self-Regulatory Model of Antiretroviral Adherence (Reynolds, 2003).

## Methodology

This two-group, randomized clinical trial examined adherence rates in 48 adults started on a new oral chemotherapeutic agent. The study explored the effectiveness of a tailored adherence intervention that was based on the particular needs of patients and suggested individualized strategies to overcome barriers to adherence.

The primary aim of the study was to test the effectiveness of a tailored protocol in promoting adherence to oral chemotherapeutic agents in adults receiving a new oral chemotherapeutic agent. The hypothesis was as follows: Adults diagnosed with cancer who participate in the experimental tailored adherence intervention will show significantly greater adherence to oral chemotherapy at 2 and 4 months, as measured by self-report and pharmacy fill rates, than a matched control group who receive only standard chemotherapy education.

In addition, the study had two exploratory aims: (1) Examine adherence to oral chemotherapeutic agents over time at 2 and 4 months, and (2) examine the effects of age, gender, and depression on adherence to oral chemotherapeutic agents.

## Setting and Sample

Subjects were recruited from the Duke Comprehensive Cancer Center in Durham, North Carolina, and from Duke Raleigh Hospital in Raleigh, North Carolina, a community affiliate. These sites serve cancer patients from a variety of socioeconomic and cultural backgrounds. The study was approved by the Duke Institutional Review Board.

The sample for this study was 48 adults who were scheduled to receive oral chemotherapy for the eradication or control of their cancer (breast cancer, colorectal cancer, renal cell carcinoma, hepatocellular carcinoma, multiple myeloma, or chronic leukemia). Patients diagnosed with these types of cancer were chosen because many of the current treatment regimens involve the use of targeted oral chemotherapeutic agents for several months. Inclusion criteria were (1) a diagnosis of breast cancer, colorectal cancer, renal cell carcinoma, hepatocellular carcinoma, multiple myeloma, or chronic leukemia; (2) age 18 years or older; (3) a treatment regimen that includes at least one oral chemotherapeutic agent; (4) ability to read and write English; (5) ability to give informed consent; and (6) willingness to sign a release form to have prescription refills reported by their pharmacy.

A total of 60 patients were approached, and 48 were enrolled in the trial. As 3 participants had their medications discontinued prior to the 2-month adherence measures and were dropped from the study, the analysis was based on a sample of 45 patients. The study sample was diverse, with 68.8% of patients being Caucasian. The average participant was a 59.85-year-old female with some college education (see Table 1 for more demographic information).

**Table 1:**
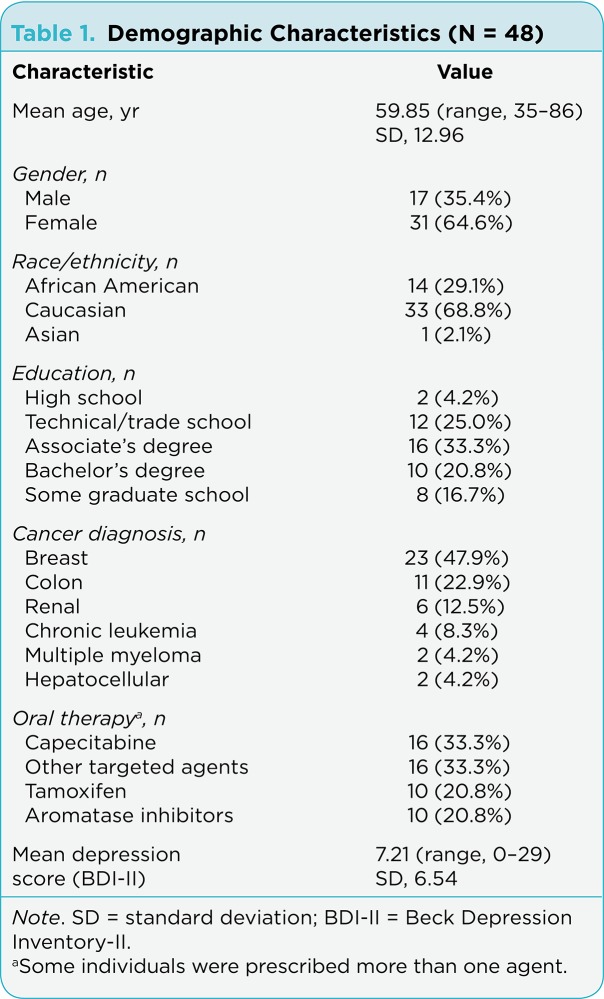
Table 1. Demographic Characteristics (N = 48)

## Procedure

Within the first week of starting a new oral chemotherapeutic agent, the research nurse spoke with those who met the study criteria via telephone or during their clinic visit. The research nurse explained the study and answered any questions. After giving informed consent, participants completed baseline instruments: a demographic information form, the Beck Depression Inventory-II (BDI-II; Beck, Steer, & Brown, 1996), and the Memorial Symptom Assessment Scale (MSAS; Portenoy et al., 1994).

Age, gender, diagnosis, and depression all have the potential to affect adherence to chemotherapeutic agents. These data were collected at baseline for both the intervention and control groups. 

Depression was measured at baseline using the BDI-II, a 21-item self-report questionnaire that asks the respondent to indicate the presence of emotional, behavioral, and physical symptoms associated with depression (Beck et al., 1996). This instrument was selected because of its established reliability and validity and known success in predicting depression in patients with cancer (D’Antonio et al., 1998). The BDI-II has high internal consistency, with á coefficients of 0.86 and 0.81 for psychiatric and nonpsychiatric populations, respectively (Beck, Steer, & Garbin, 1988).

The MSAS was developed to provide comprehensive symptom assessment information (Portenoy et al., 1994). The scale has 32 symptoms and 3 dimensions: frequency, severity, and distress. This particular scale was chosen because it measures symptoms such as skin changes and numbness in hands and feet that are commonly seen in patients receiving oral medications for chemotherapy. The MSAS has established reliability and validity with cancer patients and can be completed in 15 minutes. The instrument was completed at baseline and at 2 and 4 months. Information from the MSAS was used to guide the APN coaching intervention and to measure patient outcomes.

Following completion of the consent form and initial questionnaires, subjects were randomly assigned to receive the tailored adherence protocol or standard chemotherapy education. Randomization was conducted by the study statistician, and groups were stratified by disease.

Patients in the control group received the standard chemotherapy education provided at the cancer center. This includes a disease-specific patient education notebook and instruction on their treatment provided by a clinic nurse, the medical oncologist, or a nurse practitioner. Participants in the intervention group received the standard chemotherapy education as well. But in addition, they received a personalized assessment and a tailored intervention plan developed by an APN. Participants in the intervention group received a phone call weekly for the first month they were taking the oral chemotherapy and then twice a month for 6 months or until they completed their medication.

During the initial call, the APN discussed possible background experiences (barriers or facilitators to adherence) using the Reynolds adherence model as a guide. Questions were asked regarding illness experiences (symptoms/side effects, complexity of regimen), interactions with others (significant others, health-care providers), caregiver availability, informational resources, cognitive function, mood state, and depression. Responses to these baseline measures and the initial intervention call were used to identify specific adherence strategies that were tailored to the individual’s needs. During subsequent calls, strategies were evaluated for effectiveness and modified or reinforced as needed.

Adherence strategies can be grouped into one of three categories based on the Reynolds adherence model: knowledge strategies, behavioral skills, and affective support. To illustrate, a patient who had trouble remembering to take his medicine, particularly in the morning, was taught individualized cues. This patient might be taught to take the medication before lunch and to use a pillbox or alarm reminder system. These strategies are categorized as behavioral skills. The person’s caregiver (if available) could be involved in the discussion. In one case, a participant’s daughter was asked to help fill the weekly pillbox when she visits on Sundays. This is an example of a strategy to strengthen affective support. Both the study participants and the caregivers received information about how the medication works and why it needs to be taken daily (knowledge strategies).

A more complete example of a tailored coaching intervention is the case of a 69-year-old man with colon cancer who just started taking capecitabine twice daily. He lives alone but talks to his daughter on the phone every day. His depression assessment shows that he is not depressed, and the MSAS shows minimal side effects at this time. His other medications include taking a "thyroid pill" in the morning. Based on this background experience information, the APN recommends the following:

*Knowledge strategies*: Provide the patient with a rationale for adherence and information about side effects to expect. The patient can be advised about how to manage the side effects as opposed to not taking his medicine when side effects occur.

*Behavioral skills*: Ask the patient’s daughter to remind him about taking his medicine when she calls in the evening. The patient reported that he remembers to take his morning pill because this is more routine for him and because it coincides with his thyroid medication.

*Affective support*: Provide weekly phone call reminders, as the patient reports that this is helpful.

During a subsequent call with this participant, the APN evaluated whether the reminder call from the daughter was working. She also sent the participant a side-effect management sheet to help him manage his neuropathy, a newly reported side effect. See the Appendix for additional sample adherence strategies in each category.

## Results

Patient adherence rates were measured in both groups at 2 and 4 months using self-report and pharmacy fill rates. A research assistant, who was blind to group assignment, collected the self-report adherence and MSAS data via a telephone call to the participants in both groups. Study participants were asked: "Most of us miss doses at times. What has your experience been this past week? Were you able to take your medications as intended this past week? When did you last miss a dose? Can you recall the reason for the missed dose?" This information was used to calculate a compliance rate. The total number of doses taken per week was divided by the total number of doses prescribed.

Pharmacy records were monitored by a clinical pharmacist or nurse practitioner. Pharmacy refill records were reviewed to determine when a refill had been obtained. Medical records were reviewed to check for dose changes or any other occurrences that could account for a disruption in medication (i.e., dose reduction or surgery). The pharmacist or nurse practitioner then determined whether or not the participant had an adequate supply of medication in order to take the oral chemotherapy agent as prescribed.

In both the self-report and pharmacy refill measures at 2 and 4 months, the intervention group adherence rates were superior to the control group rates (see Table 2). Group comparisons were analyzed using chi-square tests. Due to the small sample size, none of these differences was statistically significant. Pharmacy refill rates of adherence were lower than self-reports of adherence. The differences between the intervention group and the control group suggest that for some participants, the tailored coaching intervention was beneficial in promoting adherence.

**Table 2:**
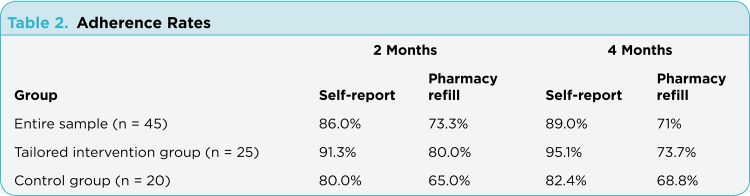
Table 2. Adherence Rates

A secondary aim of this study was to determine what the adherence rate was over time in this sample. For the entire sample, self-reports of adherence were 86.0% and 89.0% over 2 and 4 months, respectively. The percentage of individuals who had an adequate supply of medication in order to take their medication as prescribed was 73.3% at 2 months and 71% at 4 months. Table 2 provides the results for aims 1 and 2.

We found no correlation between age, gender, and depression with adherence rates to oral chemotherapeutic agents. There was one significant correlation (*(p* = .0048) between self-report of adherence and pharmacy refill rates at the 2-month time point.

An unexpected finding was that system barriers interfered with adherence in approximately 10% of participants. These are factors that were not related to the patient’s actions. Examples of system barriers include late specialty pharmacy deliveries, ordering of incompatible medications, and unclear instructions regarding when to start oral agents. All of these factors contributed to adherence rates. An example of a system barrier that occurred in this study was seen in the case of a 50-year-old woman with breast cancer. She was prescribed to take anastrozole (Arimidex) 1 mg every day and had a bone scan ordered. She was instructed to start the medication after she heard about the bone scan results and after the completion of radiation therapy. The patient was not contacted regarding the bone scan results, so she did not start the anastrozole as prescribed.

The tailored intervention was easily implemented by two APNs. Consistency in coaching between the APNs was assured through initial training with mock subjects and reliability testing every 4 months. The coaching was conducted via phone; participants were reached within 1 to 3 attempts. The phone coaching was enhanced by mutually determined phone call appointment times.

Overall, participants in the intervention group enjoyed receiving suggestions on how to take their medications and how to manage side effects. Consistent with patient-centered care, participants were told they could call their nurse coach with questions. While this occasionally happened, it was more common for participants to have a list of questions to discuss during the regularly scheduled call. A typical comment from a participant: "I’m glad you called. I can’t see why this wouldn’t be good for folks. Having someone check in on you makes sense. It’s a good chance to ask questions and make sure you’re on track. The doctors say to call, but that can be so intimidating."

Based on anecdotal findings, we would recommend implementing the tailored intervention for the initial 4 months of administration of a new oral chemotherapeutic agent. After 4 months, individuals had either figured out how to take the medication appropriately or in many cases the medications had been discontinued due to disease progression or intolerable side effects.

## Discussion

Findings regarding self-report of adherence from this study (80% to 95%) are consistent with those in the literature. This is one of the first studies to report both objective and subjective measures of adherence. In all cases, self-report rates of adherence were greater than those indicated by pharmacy refill rates. There was a significant positive correlation (*(p* = .0048) between self-report of adherence and pharmacy refill rates at the 2-month time point. This provides modest support for the validity of using self-report as an outcome measure. The positive correlation may have been a result of the relationship between the nurse coach and the patient. According to the patient-centered care concept, this relationship is the foundation to promoting individual participation regarding treatment decisions (IOM, 2001).

The major limitation of this study was the small sample size. Despite numerous strategies to boost enrollment, participant numbers remained low. The major reason potential participants gave for not enrolling in the study was that they just wanted to take their medication and not talk about it. Some individuals stated that enrolling in the study would "just remind me that I have cancer." In addition, many providers failed to refer potential participants to the study due to the belief that patients will automatically take their medication because it is prescribed and because they want to get rid of their cancer. Many providers were truly surprised when the pharmacy refill rates of 65% at 2 months and 68% at 4 months were presented to them.

Another limitation of the study was the fact that it was conducted within a single health system. The participants were recruited from both a comprehensive cancer center and an affiliated community hospital, so they were representative of cancer patients in the region. For the results to be generalizable, however, future studies should be conducted at multiple sites involving various regions of the country.

The findings of this study provide direction for clinical practice. It seems that for some patients, a pamphlet and instructions in the clinic setting may not be sufficient to promote adherence. The coaching intervention can be implemented as part of routine care for patients taking oral chemotherapy agents. Patients who request additional support or who have risk factors for decreased adherence can be targeted to receive nurse coaching. While this may involve more clinician time, in some cases the nurse coaches identified critical barriers to adherence and were able to intervene. One patient was going to stop taking her medication due to severe hot flashes. The nurse coach suggested some diet alterations and changing the time when she took the medication. The nurse coaches were able to facilitate timely pharmacy deliveries and prevent medication interactions. Using nurse coaches to promote adherence helps patients manage symptoms and ensures that medications are taken safely.

## Conclusion

This study was innovative from both patient-centered care and nursing care perspectives. It employed a tailored approach to promoting chemotherapy adherence in an oncology population. Both subjective and objective measures of adherence were used. Maximizing adherence to oral chemotherapy agents can have many positive outcomes, but the most important is improvement in overall survival and life expectancy.
